# Arginase-II negatively regulates renal aquaporin-2 and water reabsorption

**DOI:** 10.1096/fj.201701209R

**Published:** 2018-05-02

**Authors:** Ji Huang, Jean-Pierre Montani, François Verrey, Eric Feraille, Xiu-Fen Ming, Zhihong Yang

**Affiliations:** * Division of Physiology, Department of Medicine, Cardiovascular and Aging Research, University of Fribourg, Fribourg, Switzerland;; † Kidney Control of Homeostasis, National Center of Competence in Research, Zurich, Switzerland;; ‡ Institute of Physiology, University of Zurich, Zurich, Switzerland;; § Department of Cell Biology and Metabolism, University of Geneva, Geneva, Switzerland

**Keywords:** collecting duct, copeptin, vasopressin, urine, osmolality

## Abstract

Type-II l-arginine:ureahydrolase, arginase-II (Arg-II), is abundantly expressed in the kidney. The physiologic role played by Arg-II in the kidney remains unknown. Herein, we report that in mice that are deficient in Arg-II (Arg-II^−/−^), total and membrane-associated aquaporin-2 (AQP2) protein levels were significantly higher compared with wild-type (WT) controls. Water deprivation enhanced Arg-II expression, AQP2 levels, and membrane association in collecting ducts. Effects of water deprivation on AQP2 were stronger in Arg-II^−/−^ mice than in WT mice. Accordingly, a decrease in urine volume and an increase in urine osmolality under water deprivation were more pronounced in Arg-II^−/−^ mice than in WT mice, which correlated with a weaker increase in plasma osmolality in Arg-II^−/−^ mice. There was no difference in vasopressin release under water deprivation conditions between either genotype of mice. Although total AQP2 and phosphorylated AQP2-S256 levels (mediated by PKA) in kidneys under water deprivation conditions were significantly higher in Arg-II^−/−^ mice compared with WT animals, there is no difference in the ratio of AQP2-S256:AQP2. In cultured mouse collecting duct principal mCCD_cl1_ cells, expression of both Arg-II and AQP2 were enhanced by the vasopressin type 2 receptor agonist, desamino-*d*-arginine vasopressin (dDAVP). Silencing Arg-II enhanced the expression and membrane association of AQP2 by dDAVP without influencing cAMP levels. In conclusion, *in vivo* and *in vitro* experiments demonstrate that Arg-II negatively regulates AQP2 and the urine-concentrating capability in kidneys *via* a mechanism that is not associated with the modulation of the cAMP pathway.—Huang, J., Montani, J.-P., Verrey, F., Feraille, E., Ming, X.-F., Yang, Z. Arginase-II negatively regulates renal aquaporin-2 and water reabsorption.

Kidneys play a critical role in the control of body water homeostasis. Aquaporin-2 (AQP2) is a water channel protein that is abundantly expressed in principal cells of the connecting tubules and collecting ducts and is physiologically regulated by antidiuretic hormone, also known as arginine-vasopressin (AVP) (1). Under basal conditions of absence or low concentrations of AVP, most AQP2 is stored in intracellular storage vesicles (2). Under conditions of increased blood osmolality and/or decreased blood volume, AVP is released from the neurohypophysis and enhances expression and translocation of AQP2 from intracellular vesicles to the apical cell membrane *via* the vasopressin receptor subtype, vasopressin type 2 (V_2_) (3). AQP2 in the apical membrane promotes water permeability, which leads to water reabsorption, concentrated urine, and, ultimately, a decrease in blood osmolality and/or an increase in blood volume (1). Renal collecting duct–specific ablation of AQP2 causes a severe urinary-concentrating defect with 10-fold increased urine production and decreased urinary osmolality, which demonstrates an essential role of collecting duct AQP2 in the control of urine concentration and body water homeostasis (4). Accordingly, dysregulation of AQP2 has been linked to a number of renal disorders that are characterized by body-water balance disturbances, including hereditary nephrogenic diabetes insipidus, lithium-induced nephrogenic diabetes insipidus, acute and chronic renal failure, ureteral obstruction, and nephrotic syndrome, *etc.* (1).

Arginase is a manganese-containing hydrolase that metabolizes l-arginine to urea and l-ornithine (5). Two major isoforms of arginase [*i.e.*, cytoplasmic arginase-I (Arg-I) and mitochondrial arginase-II (Arg-II)] have been identified which are encoded by 2 separate genes (6). Arg-I is expressed most abundantly in the liver, where its primary function is the detoxification of ammonia *via* the urea cycle, whereas Arg-II is widely expressed in extrahepatic tissues, most abundantly in the kidney (7). Although enhanced Arg-II expression/activity has been reported in pathologic conditions to mediate renal injury by reducing the bioavailability of the vasodilator, NO, by competing with eNOS for the common substrate, l-arginine (8–10), the physiologic role of Arg-II in the kidney remains unknown. Herein, we provide the first evidence to our knowledge of a physiologic function of Arg-II in the negative regulation of AQP2 expression and function in the collecting ducts of the kidney.

## MATERIALS AND METHODS

### Reagents

Reagents were purchased or obtained from the following sources: desamino-*d*-arginine vasopressin (dDAVP; desmopressin, V1005), insulin (I1882), dexamethasone (D8893), selenium (S9133), transferrin (T1428), triiodothyronine (T5516), and mouse epidermal growth factor (E4127) were purchased from MilliporeSigma (Burlington, MA, USA); DMEM-F12 (31331) was from Thermo Fisher Scientific (Waltham, MA, USA); rabbit Ab against Arg-II (sc-20151) and goat Ab against AQP2 (sc-9882) were from Santa Cruz Biotechnology (Dallas, TX, USA); PKA inhibitor (14-22 amide) was from MilliporeSigma; and rabbit Ab against AQP2, Na^+^-Cl^−^ cotransporter (NCC), and Na^+^-K^+^-2Cl^−^ cotransporter (NKCC2) were kindly provided by Prof. Johannes Loffing (University of Zurich, Zurich, Switzerland) (11, 12). Ab against tubulin was from MilliporeSigma. Rabbit Ab against pSer256-AQP2 (ab111346) and mouse mAb against Na^+^-K^+^-ATPase (ab7671) were from Abcam (Cambridge, United Kingdom). IRDye 800–conjugated affinity-purified goat anti-rabbit IgG F(c) was purchased from Bioconcept (Alschwil, Switzerland), Alexa Fluor 680–conjugated goat anti-mouse IgG (H + L) secondary Ab (A21057), Alexa Fluor 488–conjugated goat anti-rabbit IgG (H + L) secondary Ab (A-11008), and Alexa Fluor 546–conjugated donkey anti-goat IgG (H + L) secondary Ab (A-11056) were from Thermo Fisher Scientific.

### Cell culture

Mouse mCCD_cl1_ cells were kindly provided by Prof. Edith Hummler (University of Lausanne, Lausanne, Switzerland) (13) and were cultured in plastic plates in modified DMEM (DMEM/Ham’s F-12, 1:1 v/v) that was supplemented with 2% fetal calf serum, 100 U penicillin and streptomycin, 60 nM sodium selenite, 5 µg/ml transferrin, 50 nM dexamethasone, 1 nM triiodothyronine, 5 ng/ml epidermal growth factor, and 5 µg/ml insulin. For serum starvation, cells were incubated in DMEM:F-12 that was supplemented with 100 U penicillin and streptomycin, 60 nM sodium selenite, and 5 µg/ml transferrin overnight.

### Recombinant adenovirus

Recombinant adenovirus (rAd)/U6-LacZ^shRNA^ and rAd/U6-Arg-II^shRNA^ were generated and characterized as described by Ming *et al*. and Yepuri *et al*. (14, 15).

### Adenoviral transduction of mCCD_cl1_ cells

For adenoviral transduction of mCCD_cl1_ cells, cells were seeded at a density of 0.5 × 10^5^ cells/cm^2^. Transduction of the cells by rAd was performed as described by Ming *et al*. (14). After 2 or 3 d, when cells reached confluence, they were transduced with rAd at titers of 50–100 multiplicities of infection and cultured in complete medium for 2 d. At d 2 after transduction, transduced cells were serum starved for 12 h before starting experiments.

### Animals

Arg-II^−/−^ mice were kindly provided by Dr. William O’Brien (Baylor College of Medicine, Houston, TX, USA) (16) and backcrossed to C57BL/6 for more than 10 generations (14). Genotyping was performed by RT-PCR as described by Shi *et al*. (16). Wild-type (WT) and Arg-II^−/−^ offspring from hetero/hetero cross were interbred to obtain WT and Arg-II^−/−^ mice, respectively, for experiments. Mice age 5 mo, with or without water deprivation (WD), were sacrificed after anesthesia with xylazine (10 mg/kg body weight, i.p.) and ketamine (100 mg/kg body weight, i.p.) for blood collection and isolation of kidney. The left kidney was snap frozen in liquid nitrogen and kept at −80°C until use. The right kidney was fixed with 3.7% paraformaldehyde and embedded in paraffin for immunofluorescence staining. Animal work was approved by the Ethical Committee of Veterinary Office of Fribourg Switzerland (2013_08_FR) and performed in compliance with guidelines on animal experimentation at our institution.

### Metabolic cage experiments

WT and Arg-II^−/−^ mice were maintained in standard animal house conditions with a 12-h light/dark cycle. Before metabolic cage experiments, mice were acclimatized individually for 3 d (8 h/d in metabolic cage). After acclimation, mice were put in a metabolic cage (Indulab, Gams, Switzerland) individually for 12 h from 7:00 pm to 7:00 am during a night-time period (active phase) without food to avoid any food contamination in collected urine. According to the Ethical Committee of Veterinary Office of Fribourg, only 12 h of WD under food withdraw was permitted. Two sets of 12-h metabolic cage experiments were performed on each mouse: free access to water (basal condition), and water deprivation (WD). The interval between the 2 sets of metabolic cage experiments on the same mice was at least 3 d to allow for recovery from any stress response induced by the experimental procedure. Water intake was measured and urine was collected. In another series of experiments, blood was collected from mice with or without WD for the same 12 h housed in normal cages but with free access to food. Urine and blood osmolality were measured by using a Fiske One-Ten Micro-Sample Osmometer (IG Instrumenten-Gesellschaft, Zurich, Switzerland). We analyzed plasma Na^+^ concentration using an IL 943-Flame Photometer (Instrumentation Laboratory, Bedford, MA, USA).

### Measurement of plasma copeptin

Blood plasma that was collected from mice with or without WD was used to determine copeptin, the C-terminal part of provasopressin, which is stable in blood plasma and used as a surrogate marker for vasopressin release (17). Copeptin was measured by ELISA (LS-F7101; LifeSpan BioSciences, Seattle, WA, USA) according to the manufacturer’s instructions.

### Preparation of crude membrane fraction

Crude membrane fractions were prepared from whole kidney as described by Marples *et al*. (18). In brief, frozen kidney was ground to a fine powder using a mortar and pestle in a liquid nitrogen bath. A portion of fine powder was then homogenized in 300 µl of ice-cold sucrose buffer (250 mM sucrose and 10 mM triethanolamine, pH 7.6) that contained protease inhibitor cocktail (B14002; Biotool, Munich, Germany) with XENOX-Motorhandstück MHX homogenizer on ice. Homogenate was centrifuged in a Sorvall Legend Micro 17R at low-speed 4000 *g* for 10 min at 4°C to remove nuclei and cell debris. Fifty microliters of supernatant was reserved as total kidney lysates. The supernatant was then centrifuged at 17,000 *g* for 20 min. The resultant pellet that contained plasma membranes was washed 3 times and resuspended in 50 μl of sucrose buffer as a crude membrane fraction. The supernatant was used as the nonsurface membrane fraction. Protein concentration was determined with Bio-Rad DC Protein Assay Kit according to the manufacturer’s instructions (Hercules, CA, USA).

### Isolation of inner medulla

WT mice under either basal or WD conditions for 24 h were euthanized after anesthesia with xylazine (10 mg/kg body weight, i.p.) and ketamine (100 mg/kg body weight, i.p.). Kidneys were harvested and kept in ice-cold Krebs-Ringer buffer. After removal of perinephric fat, kidneys were sectioned along the anterior-posterior axis and the white color region of the kidney as inner medulla (IM) was separated from the rest of the kidney. Both parts of the kidney were then snap frozen in liquid nitrogen, then homogenized in ice-cold sucrose buffer as mentioned above.

### Real-time quantitative RT-PCR

Total RNA was extracted from mCCD_cl1_ cells with Trizol Reagent (Molecular Research Center, Cincinnati, OH, USA) according to the supplier’s protocol. mRNA expression was evaluated by 2-step real-time quantitative RT-PCR (qRT-PCR) analysis as described by Ming *et al*. (14). mRNA expression levels of Arg-II and AQP2 were normalized to the reference gene glyceraldehyde 3-phosphate dehydrogenase (GAPDH). Primers used for detection of mouse Arg-II, AQP2, and GAPDH were as follows: mArg-II, forward 5′-CCCCTTTCTCTCGGGGACAGAA-3′, reverse 5′-GAAAGGAAAGTGGCTGTCCA-3′; mAQP2, forward 5′-CTTCCTTCGAGCTGCCTTC-3′, reverse 5′-CATTGTTGTGGAGAGCATTGAC-3′; mGAPDH, forward 5′-ACCCAGAAGACTGTGGATGG-3′, reverse 5′-ACACATTGGGGGTAGGAACA-3′.

### Immunoblotting analysis

Whole-kidney lysates, crude membrane fractions, nonsurface membrane fractions, renal inner medulla, and kidney tissue without inner medulla homogenates were prepared as described above. Linear range was determined for each protein loading in immunoblotting as described by McDonough *et al*. (19). Lysates that contained equal amounts of protein were heated at 37°C for 15 min in Laemmli buffer and separated by 10% SDS-PAGE, then transferred to PVDF membranes. Resultant membranes were blocked with PBS-Tween 20 that was supplemented with 5% nonfat dry milk [Tris-buffered saline/Tween 20 that was supplemented with 3% bovine serum albumin (BSA) for the detection of pSer256-AQP2], then incubated with the corresponding primary Ab (4°C overnight) with gentle agitation. Dilutions of each primary Ab were presented in Supplemental Table 1. Blots were then further incubated with a corresponding anti-mouse (Alexa Fluor 680 conjugated) or anti-rabbit (IRDye 800 conjugated) secondary Abs. Signals were visualized by using an Odyssey Infrared Imaging System (Li-Cor Biosciences, Lincoln, NE, USA). Quantification of signals was performed by using Li-Cor Image Studio Software.

### Immunofluorescence staining and confocal microscope imaging

For coimmunofluorescence staining of AQP2 and Arg-II, kidneys from WT and Arg-II^−/−^ mice were isolated and fixed with 3.7% paraformaldehyde and embedded in paraffin. After deparaffinization in xylene, hydration in ethanol, and antigen retrieval in Tris-EDTA buffer (10 mM Tris base, 1 mM EDTA, 0.05% Tween-20, pH 9.0) in a pressure cooker, paraffin-embedded sections (5 μm) were blocked with 10% BSA in PBS for 1 h, then incubated with goat anti-AQP2 Ab at 4°C overnight and subsequently with fluorescence-labeled donkey anti-goat IgG (H + L) at room temperature for 2 h. Renal sections were then blocked again with PBS that contained 1% BSA and 10% goat serum for 1 h and incubated with rabbit anti–Arg-II Ab at 4°C overnight and subsequently with fluorescence-labeled goat anti-rabbit IgG (H + L) at room temperature for 2 h, followed by counterstaining with 300 nM DAPI for 3 min. This protocol avoids cross-reaction among secondary Abs. Negative control for Arg-II staining was performed by using IgG instead of anti–Arg-II as primary Ab. Immunofluorescence signals were visualized under a Leica DIM6000 confocal microscope. The same procedure was applied for coimmunostaining of mCCD_cl1_ cells cultured on a coverslip.

### cAMP assay

mCCD_cl1_ cells were cultured as described and incubated with dDAVP (10^−8^ M) for 0.5, 4, or 24 h. During the last 30 min, 0.5 mM of phosphodiesterase inhibitor, 3-isobutyl-1-methylxanthine (MilliporeSigma), was added. Intracellular cAMP levels were measured by using the BioTrak EIA system (RPN2251; GE Healthcare, Glattbrugg, Switzerland) according to the manufacturer’s instructions. Analysis of cAMP levels was performed in duplicate.

### Statistical analysis

Statistical analysis was performed as described by Ming *et al*. (14). In brief, we used the Kolmogorov-Smirnov test to first determine whether the data deviated from gaussian distributions. For normally distributed values, we performed statistical analysis with the Student’s *t* test for unpaired observations or ANOVA with Tukey test, and data are given as means ± sem. For non–normally distributed values, we performed nonparametric statistical analysis with the Mann-Whitney test or the Kruskal-Wallis test with Dunn’s multiple-comparison posttest. Values of *P* ≤ 0.05 were considered statistically significant.

## RESULTS

### Arg-II deficiency in mice increases renal AQP2 levels

**Figure 1:**
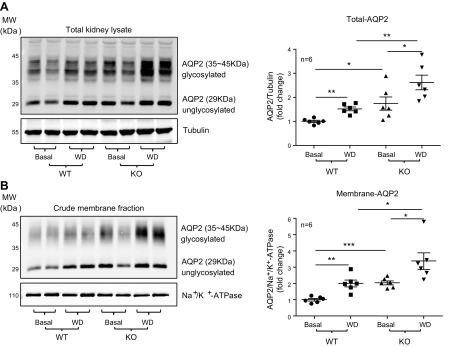
Ablation of Arg-II enhances total and membrane-associated AQP2 levels in the kidney under WD conditions in mice. Total kidney lysates and crude membrane fractions were prepared from WT and Arg-II^−/−^ mice under either basal or WD conditions for 24 h. Total kidney lysates (40 µg; *A*), and crude membrane fractions (15 μg; *B*) were loaded and subjected to immunoblotting analysis of AQP2. Tubulin and Na^+^/K^+^-ATPase were used as loading control for total kidney proteins and crude membrane proteins, respectively. Quantifications of immunoblotting signals (*n* = 6 animals in each group) are presented as dot plots in the right panels. Basal, basal condition; KO, Arg-II^−/−^. *P < 0.05, **P < 0.01, ***P < 0.001.

**Figure 2:**
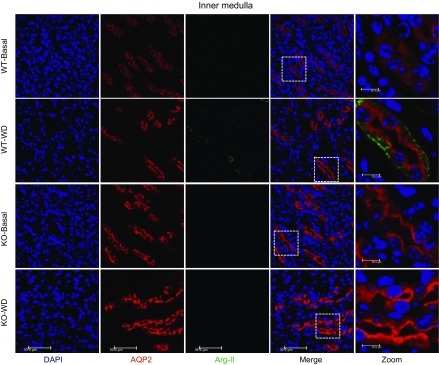
WD enhances Arg-II and AQP2 expression in the collecting ducts of inner medulla. Renal paraffin sections were prepared from WT and Arg-II^−/−^ mice under either basal or WD conditions for 24 h and subjected to immunofluorescence staining of AQP2 (red) and Arg-II (green) followed by counterstaining of the nuclei with DAPI (blue). Representative images are from 4 independent series of experiments. WT-Basal, WT-basal condition; KO-Basal, Arg-II^−/−^-basal condition; KO-WD, Arg-II^−/−^-WD.

**Figure 3:**
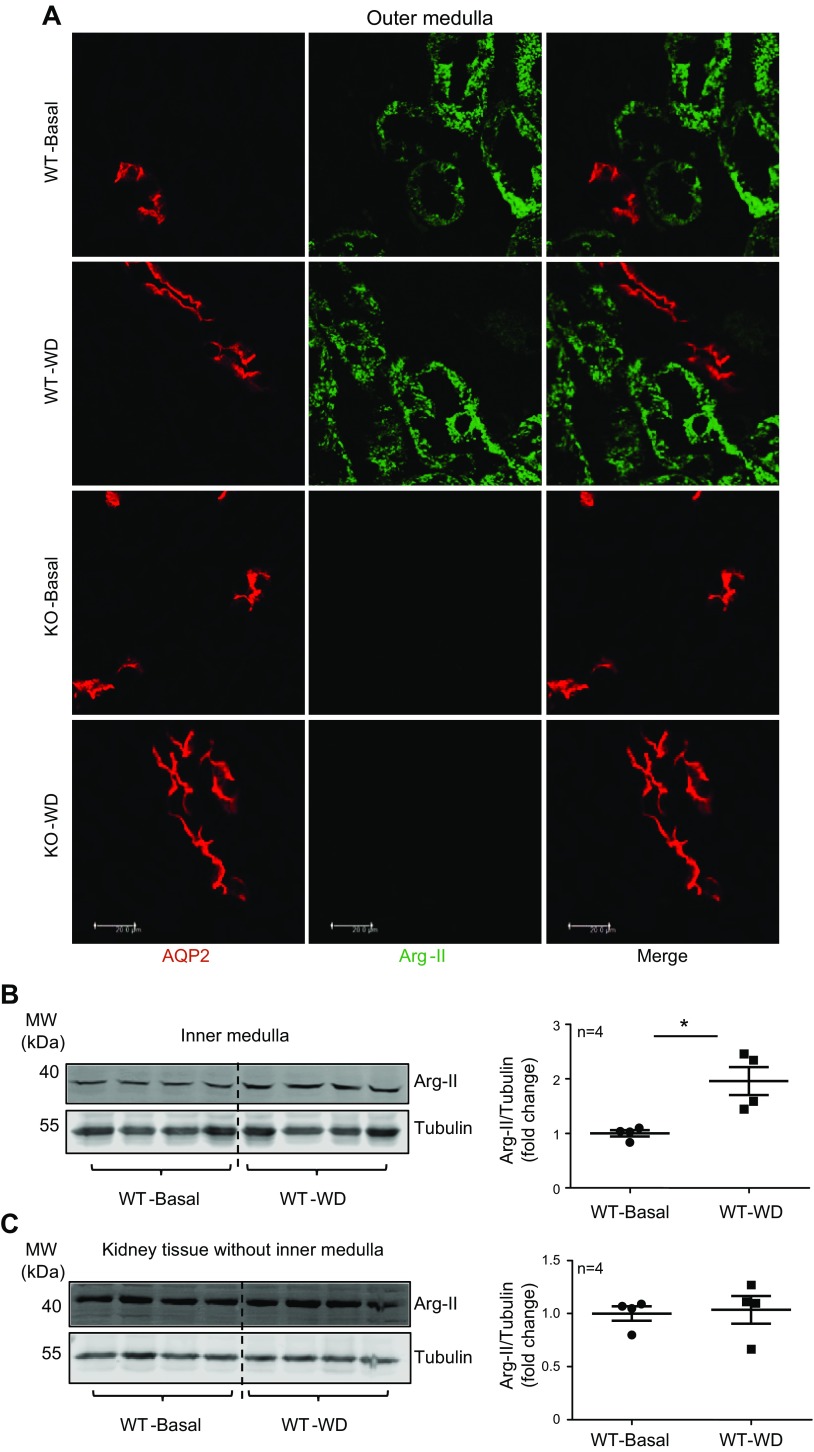
WD does not change Arg-II expression in proximal straight tubules. *A*) Kidney outer medulla paraffin sections were prepared from WT and Arg-II^−/−^ mice under either basal or WD conditions for 24 h and subjected to immunofluorescence staining of AQP2 (red) and Arg-II (green). Shown are representative images obtained from 4 independent series of mice. *B*, *C*) Immunoblotting analysis of Arg-II in inner medulla (50 µg/lane, *n* = 4 animals in each group; *B*) and kidney tissue without inner medulla (40 µg/lane, *n* = 4 animals in each group; *C*). Tubulin was used as loading control. Basal, basal condition; KO, Arg-II^−/−^. *P < 0.05.

Arg-II^−/−^ mice had higher AQP2 levels than did WT mice (**Fig. 1*A***). After WD, total and membrane-associated AQP2 levels were elevated, as expected, in WT mice, and this effect of WD was more pronounced in Arg-II^−/−^ animals (Fig. 1*A, B*). The membrane marker, Na^+^-K^+^-ATPase, was not affected by Arg-II deficiency (Fig. 1*B*), which demonstrates the specific effect of Arg-II deficiency on AQP2. In kidneys, Arg-II was expressed in AQP2^+^ collecting ducts at low levels and was enhanced by WD as shown by immunofluorescence costaining (**Fig. 2**). A higher AQP2 protein level and a more pronounced apical AQP2 accumulation were also observed upon WD in Arg-II^−/−^ mice compared with WT animals (Fig. 2). This result indicates a negative regulation of AQP2 expression and membrane association by Arg-II. Arg-II was also found abundantly and was constitutively expressed in proximal tubules as shown by our previously published studies (10, 20), which was not changed upon WD (**Fig. 3*A***). This result was further confirmed quantitatively by immunoblot analysis that demonstrated that WD significantly enhanced Arg-II levels in inner medulla but not in the rest of the kidney tissues (Fig. 3*B, C*). These results demonstrate that WD specifically regulates Arg-II expression in collecting ducts but not proximal tubules, which is in line with the observations in Figs. 2 and 3A.

### Arg-II^−/−^ augments water reabsorption and urine-concentrating capability under WD conditions

**Figure 4:**
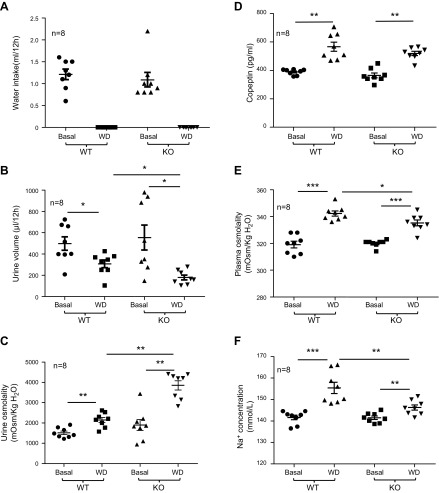
Water balance in mice under basal and WD conditions. Metabolic cage experiments were performed as described in Materials and Methods. Water intake (*A*), urine volume (*B*), urine osmolality (*C*), plasma copeptin (*D*), plasma osmolality (*E*), and plasma Na^+^ concentration (*F*) were measured in WT and KO mice under basal or WD conditions. Data are presented from 8 animals in each group. Basal, basal condition; KO, Arg-II^−/−^. *P < 0.05, **P < 0.01, ***P < 0.001.

Under the condition of free water access, no difference in water intake was observed between WT and Arg-II^−/−^ mice (**Fig. 4*A***). As expected, urine output was significantly reduced under WD conditions in both genotypes (Fig. 4*B*). Reduced urine output under WD conditions was more pronounced in Arg-II^−/−^ mice compared with WT animals (Fig. 4*B*). In accordance, urine osmolality was significantly enhanced under WD conditions in both genotypes but with a more pronounced effect in Arg-II^−/−^ mice than in WT animals (Fig. 4*C*). Copeptin, the C-terminal part of provasopressin that is stable in blood plasma and used as a surrogate marker for vasopressin release, was enhanced by WD in both WT and Arg-II^−/−^ mice (Fig. 4*D*). No difference in copeptin concentration was observed under WD conditions between the 2 genotypes. In line with the results of urine-concentrating function, plasma osmolality was enhanced under WD conditions to a greater extent in WT mice than in Arg-II^−/−^ mice (Fig. 4*E*). Plasma osmolality elevation under WD conditions in both phenotypes paralleled the changes in plasma sodium concentrations (*i.e.*, a higher plasma sodium concentration was observed in WT mice compared with Arg-II^−/−^ mice after WD; Fig. 4*F*).

Additional experiments demonstrated that the NCC level was comparable in both WT and Arg-II^−/−^ mice, which was enhanced to a similar level upon WD conditions (Supplemental Fig. 1*A*). NKCC2 level was also enhanced in WT mice upon WD conditions (Supplemental Fig. 1*B*); however, the expression of NKCC2 was variable in Arg-II^−/−^ mice and was not significantly affected by WD conditions (Supplemental Fig. 1*B*). Results indicate that the phenotype of Arg-II deficiency (*i.e.*, better water retention under WD conditions) is most likely not a result of the alteration of NCC and NKCC2, but rather because of the enhanced expression of AQP2.

### Arg-II and AQP2 are concomitantly up-regulated in collecting duct principal cells by dDAVP

We further analyzed cellular and molecular mechanisms of AQP2 regulation by Arg-II. For this purpose, a mouse collecting duct principal cell line, mCCD_cl1_, was used. In cultured cells, expression of both Arg-II and AQP2 was up-regulated by a synthetic AVP receptor subtype V_2_ agonist, dDAVP, in a concentration-dependent manner, with a maximal effect achieved at the concentration range between 10^−9^ and 10^−7^ M for 24 h (Supplemental Fig. 2*A*). Stimulation of cells by dDAVP at the concentration of 10^−8^ M up to 24 h induced a time-dependent up-regulation of both Arg-II and AQP2, starting from 6 h of stimulation (Supplemental Fig. 2*B*). In this cell line, only the unglycosylated AQP2 with an MW of 29 kD was detectable upon stimulation with dDAVP until 24 h (Supplemental Fig. 2). The concentration of 10^−8^ M and the stimulation time of 24 h were thus chosen for additional experiments. As shown in Supplemental Fig. 2*C*, the expression of both Arg-II and AQP2 was enhanced by dDAVP under this condition. Moreover, the Arg-II mRNA expression level was significantly increased by dDAVP (Supplemental Fig. 2*D*).

### Arg-II negatively modulates AQP2 expression and membrane association

**Figure 5:**
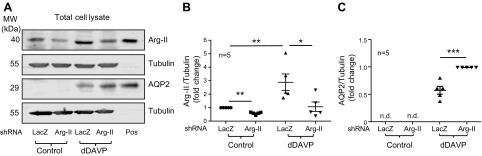
Silencing Arg-II enhances dDAVP-induced AQP2 expression. mCCD_cl1_ cells were transduced with rAd/U6-LacZ^shRNA^ as control or rAd/U6-Arg-II^shRNA^ to silence the Arg-II gene. Forty-eight hours post-transduction, cells were serum starved overnight, then incubated in the absence or presence of 10^−8^ M dDAVP for 24 h. *A*) Immunoblotting analysis of Arg-II and AQP2 was performed with total cell lysates (40 µg of total cell lysate/lane). Lysates of total kidney and inner medulla were used as positive control (pos) for Arg-II and AQP2, respectively. Tubulin served as loading control. Shown are representative blots of Arg-II and AQP2 expression. *B*, *C*) Quantifications for Arg-II (*B*) and AQP2 (*C*) immunoblotting signals are shown in dot plots. Data are presented from 5 independent experiments. N.d., not detectable. *P < 0.05, **P < 0.01, ***P < 0.001.

Additional experiments were performed to investigate a role for Arg-II in AQP2 expression in the principal cell model. Basal level and elevated expression of Arg-II stimulated by dDAVP (10^−8^ M, 24 h) were efficiently knocked down by Arg-II silencing (**Fig. 5*A, B***). There was no detectable AQP2 protein expression in cells without dDAVP stimulation (Fig. 5*A, C*). As expected, AQP2 protein expression was stimulated by dDAVP, which was further enhanced by Arg-II silencing (Fig. 5*A, C*). Changes in AQP2 protein levels paralleled changes in mRNA levels (Supplemental Fig. 6). Furthermore, AQP2 levels in crude membrane fractions and in nonsurface membrane fractions, as well as the ratio of crude membrane to nonsurface membrane–associated AQP2 were augmented by dDAVP (Supplemental Fig. 3). The membrane-associated AQP2 level was further enhanced when Arg-II was silenced (Supplemental Fig. 3*A*, *B*), which confirms our observation in the *in vivo* mouse model. Although silencing Arg-II did not further enhance the nonsurface membrane–associated AQP2 level (Supplemental Fig. 3*A*, *B*), it augmented the ratio of AQP2 in crude membrane *vs.* nonsurface membrane fractions (Supplemental Fig. 3*C*). As the crude membrane fraction is not pure plasma membrane and contains membrane of other organelles, immunofluorescence staining was performed to verify that the increase in AQP2 levels upon Arg-II silencing was indeed accumulated at the cell plasma membrane region (Supplemental Fig. 4).

### Arg-II deficiency does not affect cAMP pathway

**Figure 6:**
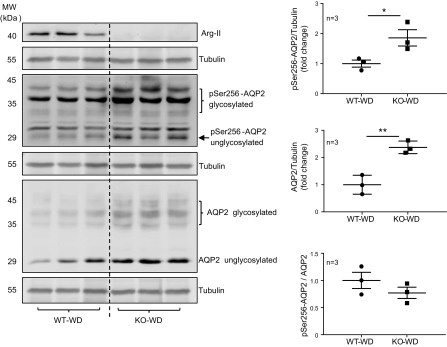
WD-induced AQP2 Ser256 phosphorylation is enhanced in Arg-II^−/−^ mice. Total kidney lysates were prepared from WT and Arg-II^−/−^ mice under WD conditions for 24 h. Total kidney lysates (40 μg) were loaded and subjected to immunoblotting analysis of Arg-II, pSer256-AQP2, and AQP2. Tubulin was used as loading control. Quantifications of immunoblotting signals (*n* = 3 animals in each group) and the ratio of pSer256-AQP2 to AQP2 are presented as dot plots in the right panels. KO-WD, Arg-II^−/−^-WD. *P < 0.05, **P < 0.01.

**Figure 7:**
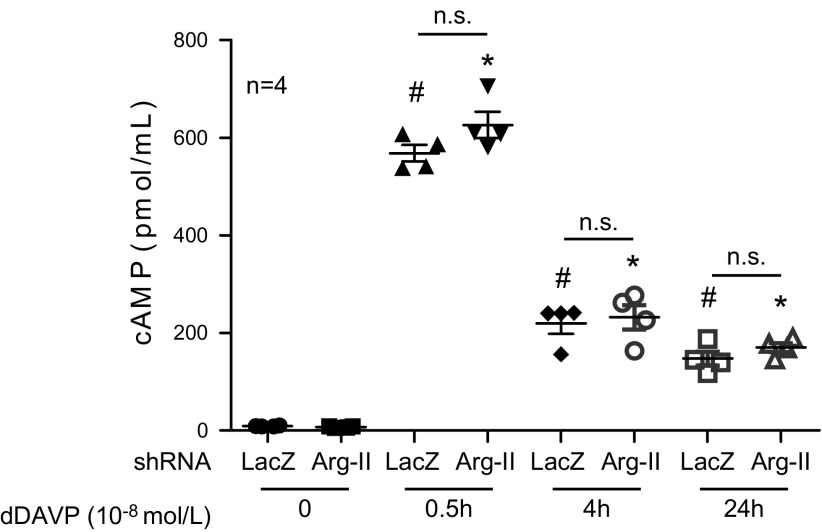
No effect of Arg-II silencing on cAMP stimulated by dDAVP was noted. There was no significant difference in the elevation of cAMP concentration in response to dDAVP after knockdown of Arg-II in principal cells. mCCD_cl1_ cells were plated onto 96-well plates and grown to confluence, then transduced with rAd/U6-LacZ^shRNA^ as control or rAd/U6-Arg-II^shRNA^ to silence Arg-II gene. Forty-eight hours post-transduction, cells were serum starved overnight, then incubated with or without 10^−8^ M dDAVP for the last 0.5, 4, or 24 h. During the last 30 min, 0.5 mM of the phosphodiesterase inhibitor, 3-isobutyl-1-methylxanthine (IBMX), was added. Cells were then lysed and intracellular cAMP was measured. N.s., not significant. *P < 0.001 compared with LacZ^shRNA^ without dDAVP, ^#^*P* < 0.001 compared with Arg-II^shRNA^ without dDAVP.

**Figure 8:**
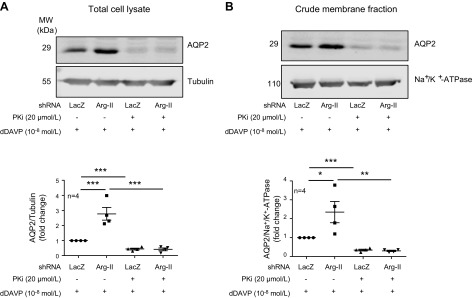
Inhibition of PKA prevents the augmentation of dDAVP-induced AQP2 by Arg-II silencing. mCCD_cl1_ cells were transduced with rAd/U6-LacZ^shRNA^ as control or rAd/U6-Arg-II^shRNA^ to silence Arg-II gene. Forty-eight hours post-transduction, cells were serum starved overnight, then incubated in the absence or presence of 10^−8^ M dDAVP for 24 h. For inhibition of PKA, cells were pretreated with 20 µM PKA inhibitor (PKi), a selective PKA inhibitor, for 1 h, then challenged continuously with PKi for 24 h. Immunoblotting analyses of AQP2 in total cell lysates (40 µg/lane; *A*) and AQP2 in crude membrane fractions (15 µg/lane; *B*) were performed. Tubulin and Na^+^/K^+^-ATPase served as loading control for total kidney lysates and crude membrane fractions, respectively. Shown are representative blots from 4 independent experiments. Data are expressed as fold change to LacZ^shRNA^ plus dDAVP group. *P < 0.05, **P < 0.01, ***P < 0.001.

As the cAMP/PKA pathway is involved in AQP2 expression and membrane association in principal cells, we investigated the effect of Arg-II deficiency on the cAMP/PKA pathway. For this purpose, levels of total AQP2 and phosphorylated AQP2-S256, mediated by PKA, in the kidney of mice under 24 h of WD conditions were analyzed. Levels of total AQP2 and AQP2-S256 were significantly higher in Arg-II^−/−^ mice compared with WT controls, without significant difference in the ratio of AQP2-S256 to AQP2 (**Fig. 6**), which suggests that the cAMP/PKA pathway is not regulated by Arg-II deficiency. Indeed, in cultured mCCD_cl1_ cells, cAMP levels stimulated by dDAVP (10^−8^ M) over 24 h were not affected by Arg-II silencing (**Fig. 7**). A similar result was also obtained with a lower concentration of dDAVP (10^−9^ M; Supplemental Fig. 5). Moreover, the stimulating effects of dDAVP on AQP2 and the enhancement by Arg-II silencing (*i.e.*, AQP2 expression and membrane association) were abolished by the PKA inhibitor (20 μM; **Fig. 8**), which demonstrates an important role for the cAMP/PKA pathway in AQP2 regulation. This pathway, however, is not modulated by Arg-II silencing.

## DISCUSSION

The current study discovered a previously undescribed physiologic role for Arg-II in the regulation of AQP2 expression and function in response to V_2_ receptor activation in the kidney. We provide both *in vitro* and *in vivo* evidence that Arg-II in collecting duct cells is up-regulated by V_2_ receptor activation in parallel with an increase in AQP2 levels, whereby Arg-II negatively regulates AQP2 expression and its membrane association, which functions as a fine-tuning mechanism of AVP-mediated water reabsorption in the kidney.

It is well documented that Arg-II is abundantly expressed in the kidney, mainly in the S3 segment of the proximal tubules (10, 21, 22). Here, we demonstrate that Arg-II is induced by the V_2_ receptor agonist in cultured collecting duct principal cells and in mouse under WD conditions, which stimulate AVP release as a physiologic response. Of note, endogenous AQP2 is not detectable in the mCCD_cl1_ cell line, but was induced by dDAVP stimulation. Of interest, Arg-II is not up-regulated in the proximal tubules but is enhanced in collecting ducts under WD conditions in mice, which indicates that Arg-II induction is dependent on V_2_ receptor activation. Up-regulation of Arg-II and AQP2 by the V_2_ receptor takes place in parallel, and Arg-II silencing in the cells *in vitro* or Arg-II deficiency in mouse reveal a more pronounced AQP2 expression and membrane association. Results demonstrate that Arg-II negatively regulates AQP2 expression and membrane association in response to V_2_ receptor activation or WD conditions. A previously published study reported that Arg-II was down-regulated in the inner medullary collecting ducts in response to dDAVP infusion in rats using proteomics analysis (23); however, down-regulation of Arg-II in that study was not validated. In our current study, we found that Arg-II is not only up-regulated by dDAVP in cultured mouse collecting duct principal cells in a dose- and time-dependent manner, but is also up-regulated in collecting duct cells upon WD in mice, a physiologic condition in which AVP release and AQP2 up-regulation are stimulated. These data thus provide *in vitro* and *in vivo* evidence for Arg-II up-regulation in collecting ducts by AVP.

Another important finding of the current study is the characterization of the role of up-regulated Arg-II in the regulation of AQP2 expression and function by the V_2_ agonist or WD. Under the condition of hypernatremia and hypovolemia, AVP is released and induces AQP2 expression and translocation from intracellular vesicles to the apical membrane in the renal collecting ducts *via* the V_2_ receptor to allow water reabsorption and the maintenance of body-water homeostasis. This process shall be terminated after correction of the water balance, which may be achieved by several mechanisms, such as a decline in AVP levels that results in AQP2 internalization (3, 24). Here, we provide evidence of an intrinsic physiologic feedback regulatory mechanism of Arg-II for AQP2 expression and function in response to V_2_ receptor activation *in vitro* and *in vivo* in a mouse model. Several experimental findings support this conclusion. First, in the cultured mouse collecting duct principal cell line, mCCD_cl1_, stimulation of the V_2_ receptor by dDAVP induces the expression and membrane association of AQP2 with concomitant up-regulation of Arg-II. An enhanced Arg-II expression in the collecting ducts is observed under WD conditions in mice in parallel with an increase in the AQP2 protein level and membrane association. Moreover, silencing Arg-II leads to enhanced expression and membrane association of AQP2 in response to dDAVP. Finally, AQP2 protein levels of both total and crude membrane fraction are higher in Arg-II^−/−^ mice than in WT control animals.

As NCC and NKCC2 could be regulated by AVP, participating in water reabsorption, we analyzed whether Arg-II deficiency could affect NCC and NKCC2 in mouse kidney. We find no significant difference in membrane-associated NCC levels at basal condition and also no difference in increased NCC levels after WD between Arg-II^−/−^ and WT mouse. Basal levels of membrane NKCC2 seem to be higher in Arg-II^−/−^ mice than in WT animals, but do not reach statistical significance. Of importance, WD significantly increases membrane NKCC2 levels in WT mice but not in Arg-II^−/−^ animals. These results likely exclude a role for NCC and NKCC2 in the better water reabsorption capability of Arg-II^−/−^ mice under WD conditions.

Of note, there is no significant difference in urine output between Arg-II^−/−^ and WT mice, although Arg-II^−/−^ mice express higher AQP2 levels than do WT mice under basal conditions; the reason is not clear. It has been speculated that the subtle enhanced water reabsorption effect by a higher AQP2 level in Arg-II^−/−^ mice under basal conditions is counteracted by other mechanisms that are involved in the regulation of urine output, which remains to be investigated; however, under WD conditions, an increase in AQP2 levels is more pronounced in Arg-II^−/−^ than in WT controls, which leads to a significant reduction in urine output and an increase in urine osmolality. As expected, WD releases vasopressin as demonstrated by the increased plasma concentration of copeptin, the stable C-terminal part of provasopressin; however, no difference in copeptin concentration is observed between the 2 genotypic mice, which suggests that Arg-II deficiency does not affect AVP release but enhances the effectiveness of AVP *via* up-regulation of AQP2 in the kidney. In support of this conclusion, plasma osmolality and sodium concentration were enhanced to a lesser extent under WD conditions in Arg-II^−/−^ mice compared with WT controls.

These results demonstrate a role for Arg-II in the control of water balance *via* negative regulation of AQP2 expression and membrane association, which represents a fine-tuning regulation of water homeostasis in response to AVP. It is presumable that the dysfunction of this mechanism may lead to pathologic changes in water balance in disease conditions. This aspect requires additional investigation.

Thus far, the best-known function of arginase, including Arg-II, in extrahepatic tissues is its l-arginine:ureahydrolase activity. By metabolizing l-arginine, arginase competes with NOS, including eNOS, iNOS, and neuronal NOS, for their common substrate l-arginine, which leads to decreased NO production in endothelial cells, macrophages, and neural tissue (25–27). In addition, arginase also exerts its biologic effects *via* its metabolite, l-ornithine, which is further utilized to synthesize l-proline and polyamines (25). Whereas l-proline is a precursor of collagen, polyamines are important substances for cell proliferation, anti-inflammatory effects in macrophages, and neuronal regeneration (25, 28–30). A possible link between NOS and AQP2 has been suggested, and evidence has been presented that NO positively modulates AQP2 expression (31, 32). The highest NOS activity in the kidney has been found in rat inner medulla collecting duct, where mRNAs of neuronal NOS, eNOS, and iNOS were detected (33). Considering that iNOS is up-regulated by AVP (23) and NO positively modulates AQP2 expression (31), a possible assumption is that AVP-induced up-regulation of Arg-II may counteract AQP2 up-regulation by inhibiting NO production. A critical question remains as to how Arg-II exerts its negative regulatory effects on the abundance of total and membrane-associated AQP2. It is well known that the cAMP/PKA pathway is involved in V_2_ receptor–mediated AQP2 expression and surface membrane translocation in principal cells (34). Results of our current study demonstrate that Arg-II deficiency regulates AQP2 in collecting ducts not by enhancing the cAMP/PKA pathway. This conclusion is supported by our findings. First, the ratio of AQP2-S256 to AQP2 is not different between Arg-II^−/−^ mice and WT controls, although levels of total AQP2 and AQP2-S256, mediated by PKA, under WD conditions are significantly higher in Arg-II^−/−^ mice compared with WT controls. Second, cAMP levels stimulated by dDAVP in cultured mCCDcl1 cells are not affected by Arg-II silencing. The fact that the stimulating effect of dDAVP on AQP2 and the enhanced effect by Arg-II silencing are abolished by PKA inhibition confirms the important role of the cAMP/PKA pathway in AQP2 regulation; however, this pathway is not modulated by Arg-II silencing. It is most likely a result of the enhanced AQP2 expression by Arg-II deficiency, as mRNA and/or total protein levels of AQP2 are elevated in Arg-II^−/−^ mice or in cultured CD cells with Arg-II silencing.

In summary, our study demonstrates that up-regulated Arg-II by V_2_ receptor activation does not interfere with the cAMP pathway that is required for AQP2 expression and membrane association. This function of Arg-II represents a physiologic negative feedback mechanism in the effects of AVP on water reabsorption. Alteration of this effect of Arg-II may be involved in body-water imbalance under pathologic conditions, which opens a new avenue to understand water imbalance under pathologic conditions.

## Supplementary Material

This article includes supplemental data. Please visit *http://www.fasebj.org* to obtain this information.

## Abbreviations

AQP2: aquaporin-2
Arg-I/II: arginase-I/II
AVP: arginine-vasopressin
BSA: bovine serum albumin
dDAVP: desamino-*d*-arginine vasopressin
GAPDH: glyceraldehyde 3-phosphate dehydrogenase
NCC: Na^+^-Cl^−^ cotransporter
NKCC2: Na^+^-K^+^-2Cl^−^ cotransporter
qRT-PCR: quantitative RT-PCR
rAd: recombinant adenovirus
V_2_: vasopressin type 2
WD: water deprivation
WT: wild type
