# Supplementary figures and images for: Arginase-II negatively regulates renal aquaporin-2 and water reabsorption
Source: FASEB J. 2018 May 2;32(10):5520–31. doi: 10.1096/fj.201701209R (PMC6405175; doi:10.1096/fj.201701209R)

A

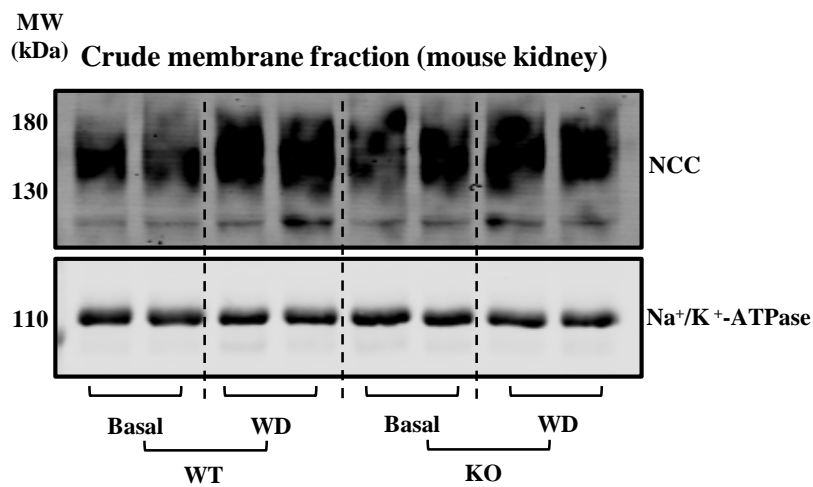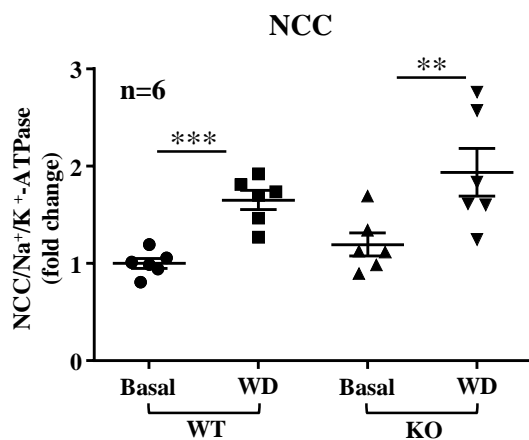

B

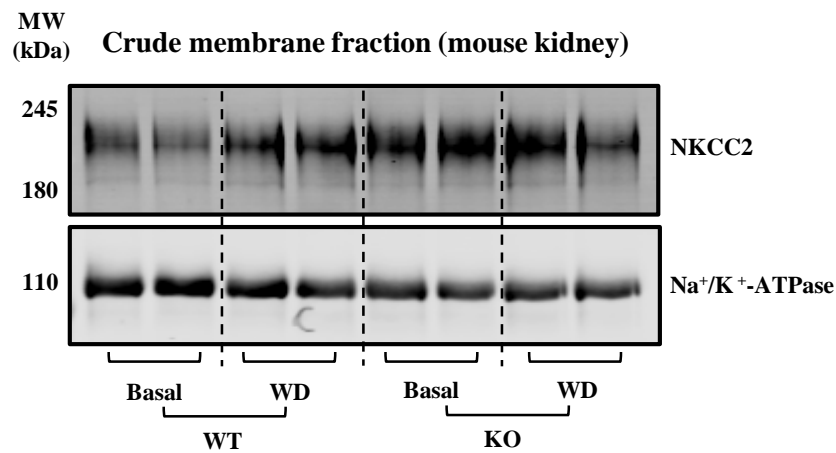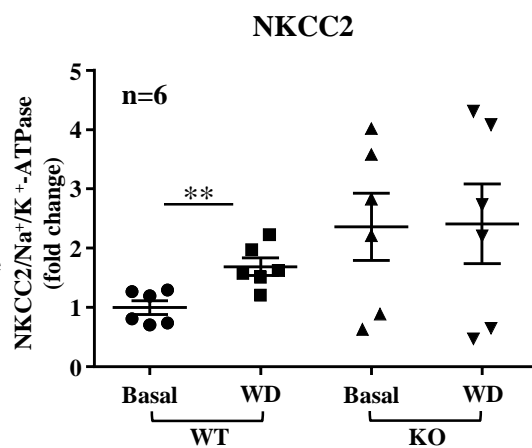

Fig. S1

Supplement: Supplementary file 1 [file fj.201701209R.sf1.pdf]

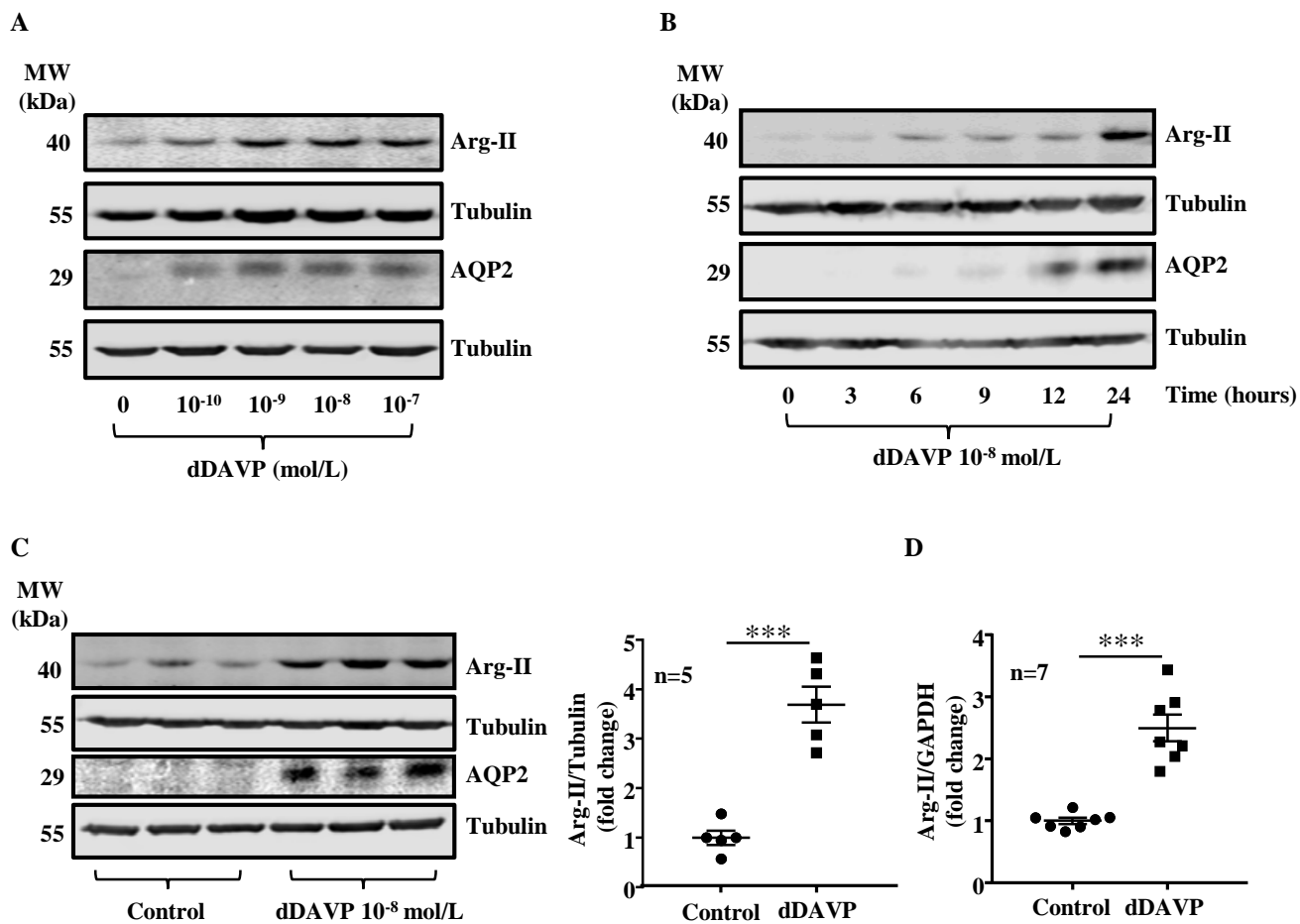

Fig. S2

Supplement: Supplementary file 2 [file fj.201701209R.sf2.pdf]

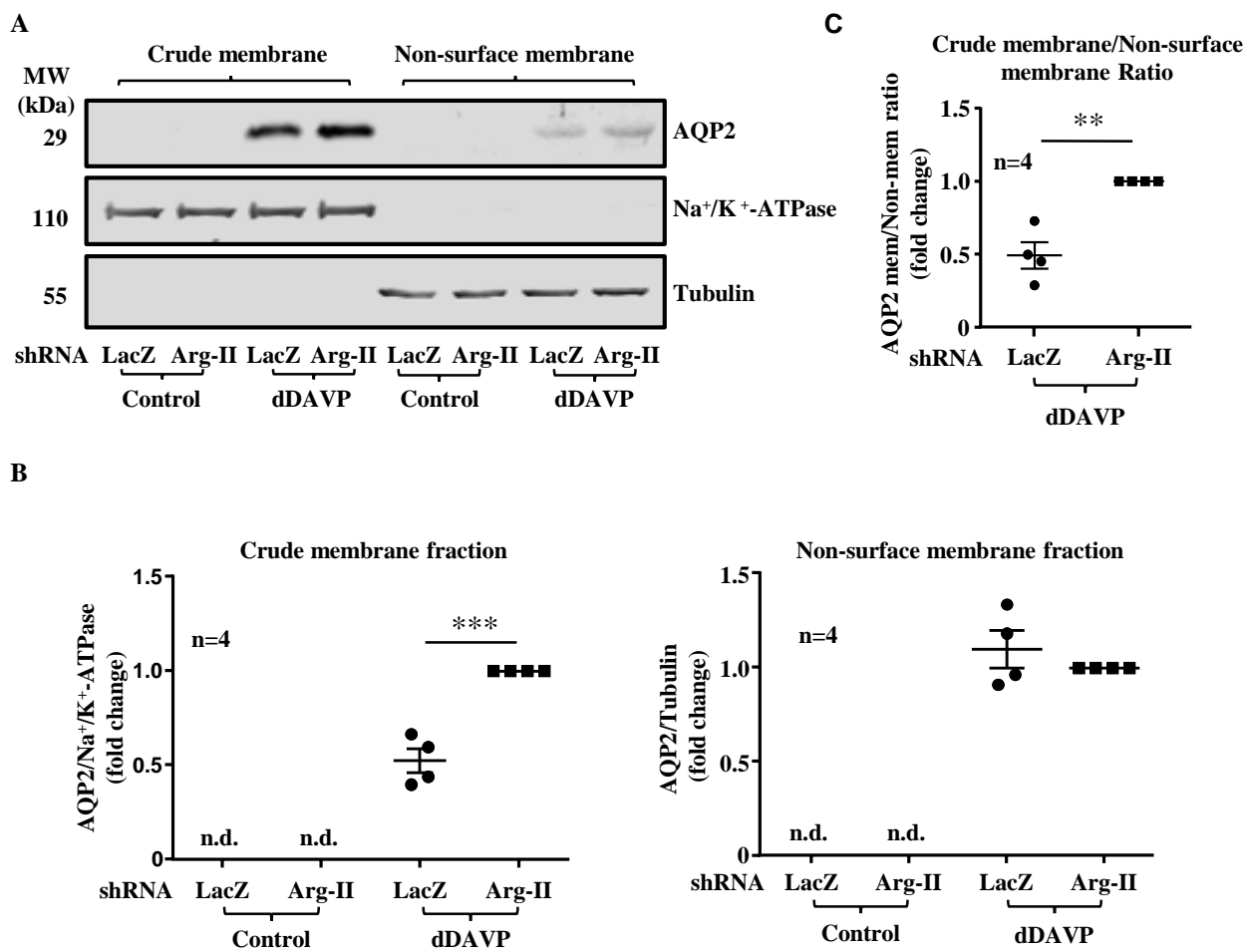

Fig. S3

Supplement: Supplementary file 3 [file fj.201701209R.sf3.pdf]

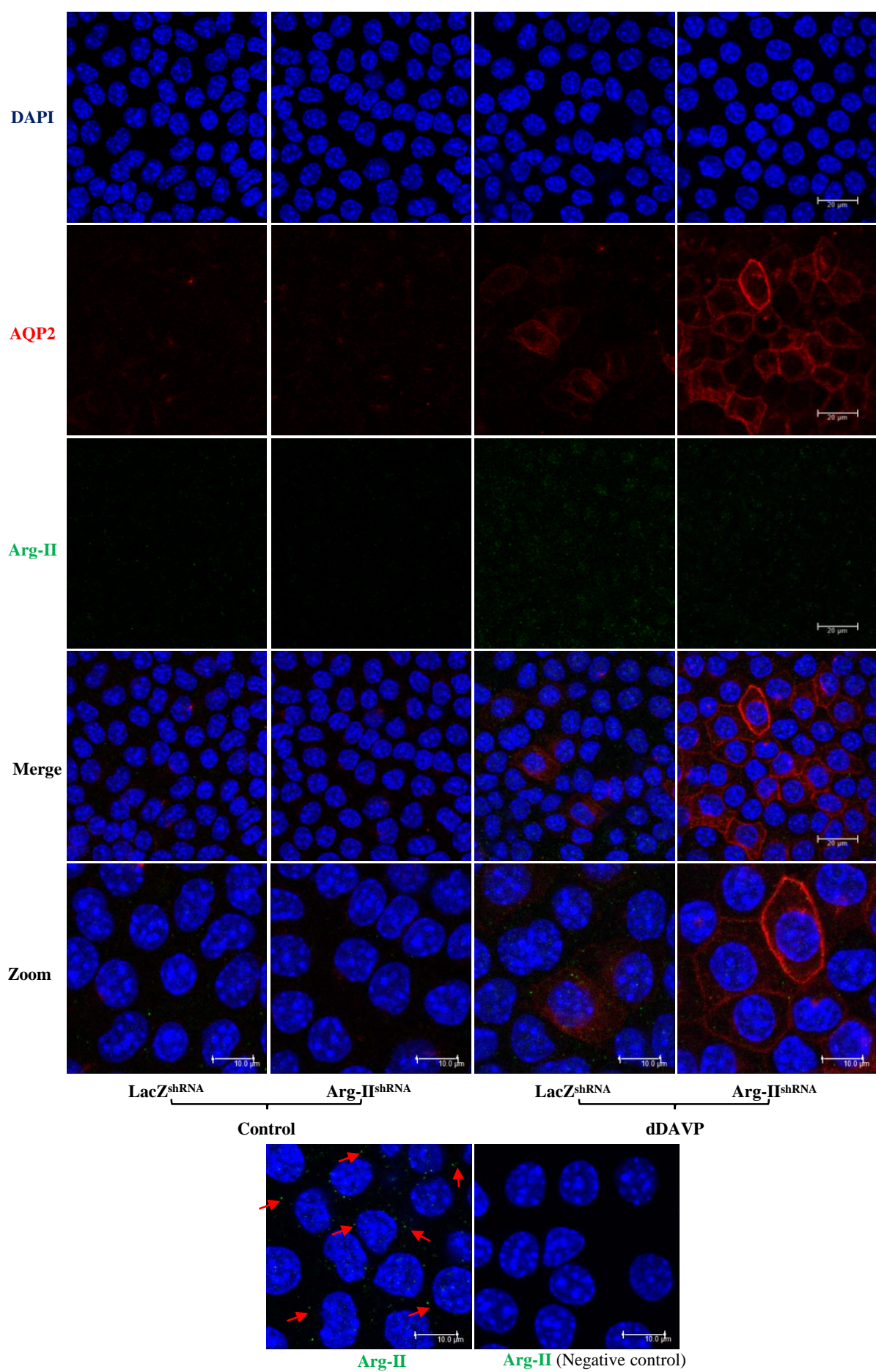

**Fig. S4**

Supplement: Supplementary file 4 [file fj.201701209R.sf4.pdf]

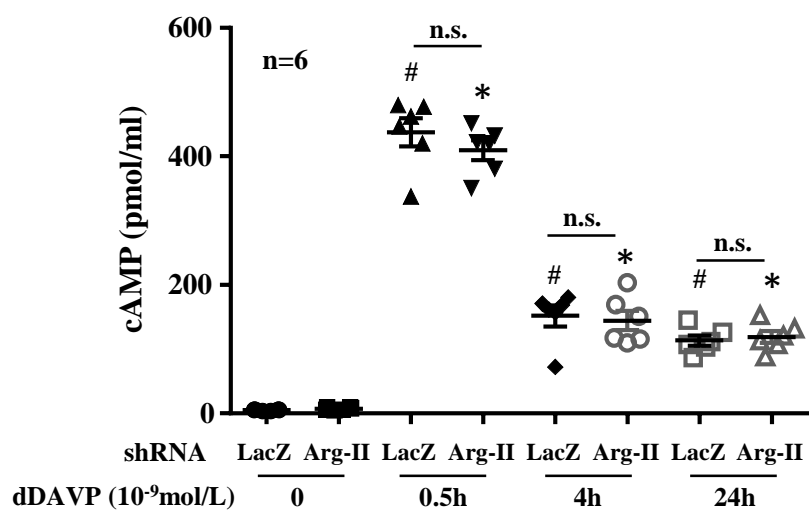

Fig. S5

Supplement: Supplementary file 5 [file fj.201701209R.sf5.pdf]

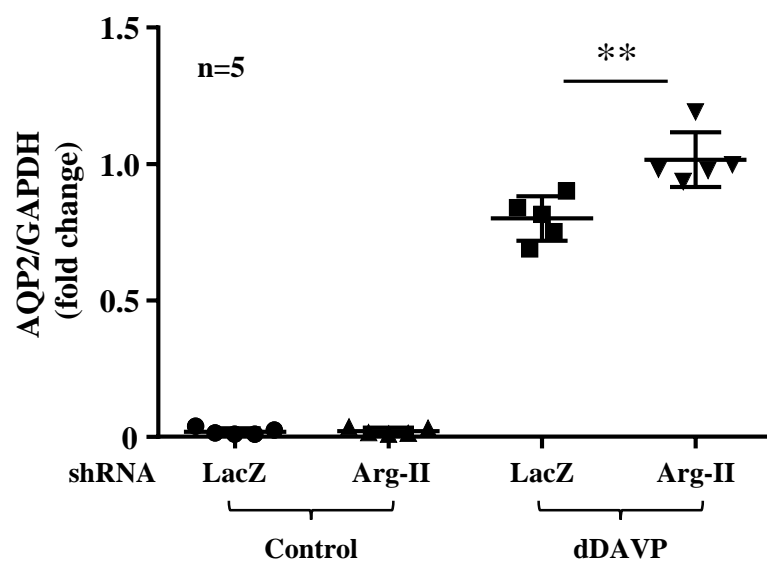

Fig. S6

Supplement: Supplementary file 6 [file fj.201701209R.sf6.pdf]

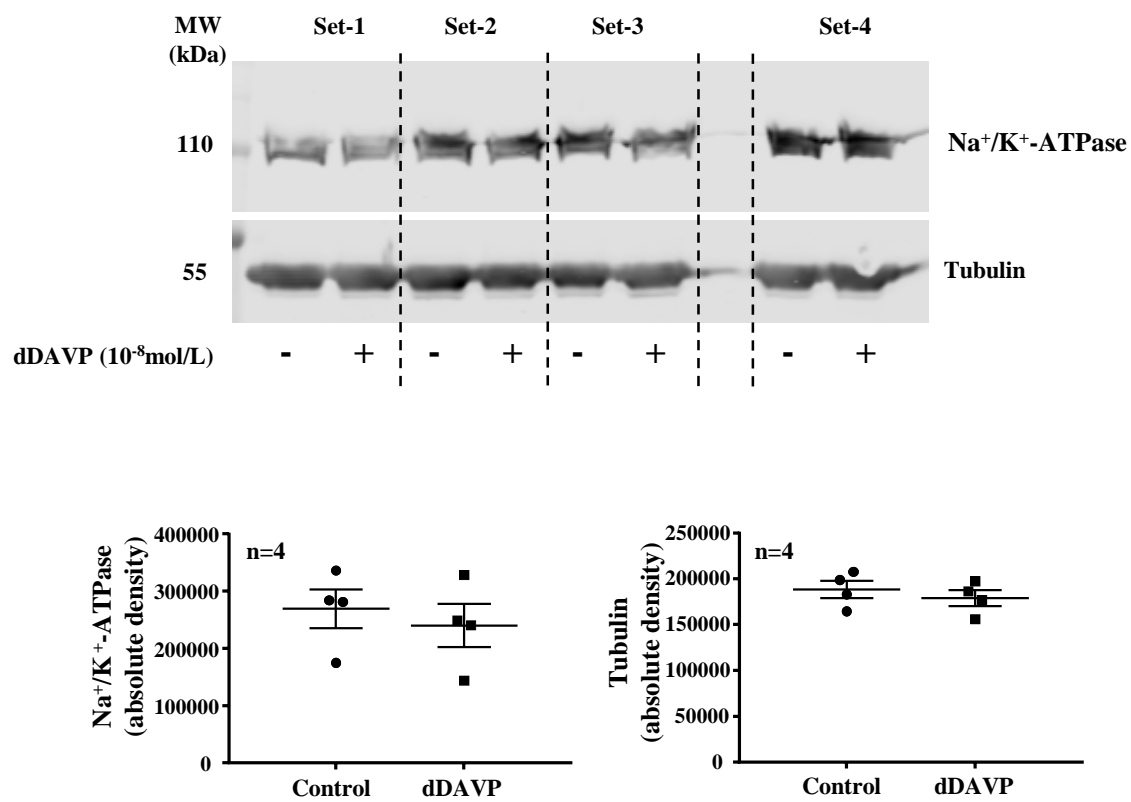

Fig. S7

Supplement: Supplementary file 7 [file fj.201701209R.sf7.pdf]
