# Supplementary material for: Arginase-II negatively regulates renal aquaporin-2 and water reabsorption
Source: FASEB J. 2018 May 2;32(10):5520–31. doi: 10.1096/fj.201701209R (PMC6405175; doi:10.1096/fj.201701209R)
Supplement: Supplementary file 8 [file fj.201701209R.st1.pdf]

Table 1. Antibodies dilution used for immunoblotting and immunofluorescence

Antibody Target	Host	Dilution
AQP2	Rabbit	WB 1:20,000
AQP2	Goat	IF 1:100
Arg-II	Rabbit	WB 1:200<br>IF 1:100
NCC	Rabbit	1:2,000
NKCC2	Rabbit	1:5,000
pSer256-AQP2	Rabbit	1:200
Tubulin	Mouse	1:50,000
Na^+/K^+-ATPase	Mouse	1:5,000
