# Supplementary material for: Arginase-II negatively regulates renal aquaporin-2 and water reabsorption
Source: FASEB J. 2018 May 2;32(10):5520–31. doi: 10.1096/fj.201701209R (PMC6405175; doi:10.1096/fj.201701209R)
Supplement: Supplementary file 9 [file fj.201701209R.sd1.docx]

**Supplemental Figure Legends**

**Fig. S1. Ablation of Arg-II does not affect NCC and NKCC2 abundance.** Immunoblotting analysis of NCC **(A)** and NKCC2 **(B)** in 15 µg of crude membrane fractions prepared from WT and Arg-II^-/-^ mice under either basal or water deprivation conditions for 24 hours. Na^+^/K^+^-ATPase was used as the loading control. Quantifications of the immunoblotting signals (n=6 animals in each group) are presented as dot plots in the right panels. MW, molecular weight; WT, wild type; KO, Arg-II^-/-^; Basal, basal condition; WD, water deprivation. , ***p*<0.01, ****p*<0.001.

**Fig. S2. dDAVP induces concomitant expression of Arg-II and AQP2 in mCCD_cl1_ cells.** **(A)** Confluent mCCD_cl1_ cells were serum-starved overnight and then incubated at 37°C in the absence or presence of various concentrations of dDAVP as indicated for 24 hours; **(B)** Time course of Arg-II and AQP2 induction by dDAVP (10^-8^ mol/L); **(C)** dDAVP (10^-8^ mol/L, 24 hours)-induced protein expression of AQP2 and Arg-II. Shown are representative blots from five independent experiments. 40 µg of total cell lysates were loaded for immunoblotting analysis. Tubulin was served as loading control. The quantification of signals for immunoblotting is shown in the dot plots. (**D**) mRNA level of Arg-II by qRT-PCR. GAPDH served as the reference for Arg-II mRNA expression. Data are expressed as fold change to control group. ****p* < 0.001.

**Fig. S3.** **Silencing Arg-II promotes AQP2 membrane association.** mCCD_cl1_ cells were transduced with rAd/U6-LacZ^shRNA^ as control or rAd/U6-Arg-II^shRNA^ to silence Arg-II gene. 48h post transduction, cells were serum-starved overnight and then incubated in the absence or presence of 10^-8^ mol/L dDAVP for 24h. Lysates of mCCD_cl1_ cells were then fractionated into crude membrane and non-surface membrane fractions. **(A)** AQP2 levels in different fractions (15 µg/lane) were analyzed by immunoblotting. Na^+^/K^+^-ATPase and tubulin were used as the loading control for crude membrane fraction and non-surface membrane fraction, respectively. **(B)** Quantifications of the immunoblotting signals. **(C)** The ratio of AQP2 expression in crude membrane vs. non-surface membrane fraction. Data are presented from four independent experiments. n.d., not detectable. ***p*<0.01, ****p*<0.001.

**Fig. S4.**  **Silencing Arg-II promotes AQP2 membrane association in response to dDAVP**. mCCD_cl1_ cells were transduced with rAd/U6-LacZ^shRNA^ as control or rAd/U6-Arg-II^shRNA^ to silence Arg-II gene. 48 hours post transduction, cells were serum-starved overnight and then treated either without or with 10^-8^ mol/L dDAVP for 24 hours. Cells were then subjected to immunofluorescence staining of AQP2 (red), Arg-II (green) and DAPI (blue). A negative control for Arg-II staining using IgG instead of anti-Arg-II as primary antibody was included (lower panel). Arrows indicate specific Arg-II staining. Shown are representative images from three independent experiments.

**Fig. S5. No effect of Arg-II silencing on cAMP stimulated by dDAVP.** No significant difference in elevation of cAMP concentration in response to dDAVP after knockdown of Arg-II in principal cells. mCCD_cl1_ cells were plated onto 96-wellplates and grown to confluence, then transduced with rAd/U6-LacZ^shRNA^ as control or rAd/U6-Arg-II^shRNA^ to silence Arg-II gene. 48 hours post transduction, cells were serum-starved overnight and then incubated with or without 10^-9^ mol/L dDAVP for the last 0.5h, 4h or 24h. During the last 30 min, 0.5mM phosphodiesterase inhibitor 3-isobutyl-1-methylxanthine (IBMX) was added. Cells were then lysed and intracellular cAMP was measured. **p*<0.001 compared with LacZ^shRNA^ without dDAVP. #*p*<0.001 compared with Arg-II^shRNA^ without dDAVP. n.s, not significant.

**Fig. S6. Silencing Arg-II enhances AQP2 mRNA level.** mCCD_cl1_ cells were transduced with rAd/U6-LacZ^shRNA^ as control or rAd/U6-Arg-II^shRNA^ to silence Arg-II gene. 48 hours post transduction, cells were serum-starved overnight and then treated either without or with 10^-8^ mol/L dDAVP for 24 hours. mRNA level of AQP2 by qRT-PCR. GAPDH served as the reference for AQP2 mRNA expression. ***p* < 0.01.

**Fig. S7. Na^+^/K^+^-ATPase is not influenced by dDAVP.** mCCD_cl1_ cells were grown to confluence, serum-starved overnight and then treated either without or with 10^-8^ mol/L dDAVP for 24 hours. 40 µg of total cell lysates were loaded for immunoblotting analysis. Tubulin was served as loading control. The quantification of signals for immunoblotting is shown in the dot plots.
